# *Candida auris* Blood stream infection- a descriptive study from Qatar

**DOI:** 10.1186/s12879-023-08477-5

**Published:** 2023-08-06

**Authors:** Junais Koleri, Hawabibee Mahir Petkar, Hussam Abdel Rahman S. Al Soub, Muna A. Rahman S. AlMaslamani

**Affiliations:** 1 Division of Infectious Diseases, Hamad Medical Corporation, PO Box 3050, Doha, Qatar; 2 Department of Microbiology, Hamad Medical Corporation, Doha, Qatar

**Keywords:** *Candida auris*, Candidemia, COVID-19, Fungemia, Blood stream infection

## Abstract

**Background:**

*Candida auris* is an emerging yeast pathogen that can cause invasive infections, particularly candidemia, in healthcare settings. *Candida auris* is characterized by resistance to multiple classes of antifungal drugs and high mortality.

**Objective:**

To describe the risk factors, clinical characteristics, antifungal susceptibility pattern and outcomes of *Candida auris* blood stream infection.

**Methods:**

We conducted a retrospective review of electronic medical records of *C. auris* fungemia cases in the facilities under Hamad Medical corporation, Qatar from 1/11/2018 to 31/7/2021. Demographic data, risk factors, antibiogram and 30-day outcome are described.

**Results:**

We identified 36 patients with *C. auris* fungemia. Most of the patients were in intensive care unit following severe COVID-19 pneumonia and had received steroids and broad-spectrum antibiotics. Most cases were central line related. Over 90% of isolates were non-susceptible to fluconazole, while amphotericin B resistance reached 85%. Factors associated with high mortality included initial SOFA score of 9 or above and absence of source control.

**Conclusion:**

Our study reveals a concerning 41.6% mortality rate within 30 days of *C. auris* candidemia. Furthermore, the prevalence of amphotericin B resistance in Qatar exceeds what has been reported in the literature necessitating further exploration. Echinocandins retains nearly 100% susceptibility and should be prioritized as the treatment of choice. These findings emphasize the need for vigilant monitoring and appropriate management strategies to combat *C. auris* infections and improve patient outcomes.

## Introduction

*Candida auris*, an emerging hospital-acquired pathogen, was first discovered in 2009 in Japan [1] and has since spread worldwide. A systematic review and meta-analysis conducted in 2020 by Chen et al. revealed a total of 4,733 cases across 33 countries [2]. Risk factors for *C. auris* colonization and infection include prolonged healthcare exposure and significant comorbidities [3]. The pathogen can colonize skin, mucosa, and various surfaces in healthcare settings. Its ability to survive on dry surfaces outside the host contributes to outbreaks in hospitals [4, 5]. It is known to spread and cause outbreaks in hospital settings owing to its ability to survive and persist outside the host on dry, nonporous surfaces for weeks [6]. *C. auris* has been isolated from a variety of clinical specimens including urine, bile, blood, wound, nose, skin, and rectum [7].

The first case of *C. auris* in Qatar was identified in November 2018 in a patient colonized in the respiratory tract [8]. Since then, *C. auris* colonization and invasive infections are increasingly reported from Qatar, with an apparent rise during the COVID-19 pandemic [9, 10]. In a previous publication from Qatar, *C. auris* was identified as a pathogen in a wide variety of invasive infections, including bloodstream infection, urinary tract infection, skin infection, and lower respiratory tract infections, especially in critically ill patients [11]. To the best of our knowledge, this is the first study from Qatar focusing solely on *C. auris* blood stream infections. By filling this knowledge gap, the study aims to inform prevention strategies, facilitate early recognition, and ensure appropriate treatment, ultimately leading to improved clinical outcomes.

## Objectives

1. To describe the risk factors, clinical characteristics, and outcomes of *Candida auris* blood stream infection.
2. To describe antifungal susceptibility patterns associated with *Candida auris.*

## Study methodology

This is a retrospective observational study. Patients from all Hamad Medical Corporation (HMC) facilities with *Candida auris* isolated from blood between 1 and 2018 to 31st July 2021 were included. Clinical and epidemiological data was extracted from the electronic healthcare records of HMC. Data were enumerated as numbers, median and percentage as appropriate. Risk factors to mortality were analyzed. Statistical analysis was done using Epi-Info™, a free data entry and statistical software developed by the Centers for Disease Control and Prevention (CDC). Candidemia was defined as the presence of at least one positive blood culture for *Candida* species in patients exhibiting clinical signs and symptoms consistent with an infection. Central line-associated candidemia was defined as laboratory-confirmed candidemia occurring in a patient with a central line either at the time of symptom onset or within 48 h prior, excluding infections originating from another site. Laboratory data including antifungal susceptibility results were extracted from the laboratory information management system. Identification of isolates was done using matrix-assisted laser desorption/ionization-time of flight (MALDI-TOF) mass spectrometry (Bruker, Billerica, U.S.A). Antifungal susceptibility testing was carried out by broth microdilution using Sensititre™ YeastOne™ YO9 AST Plate (Thermo Fisher Scientific, Carlsbad, U.S.A) or using the VITEK® AST- YS09 Test Card on the VITEK® 2 (BioMérieux, Marcy-l’Étoile, France) automated identification and susceptibility testing system. Minimum inhibitory concentrations (MICs) of antifungals were interpreted using the Clinical Laboratory Standards Institute (CLSI) interpretive criteria [12] and epidemiological cut off values (ECVs) where available [13] or the Centers for Disease Control (CDC) tentative interpretive breakpoints [14].

## Results

### Demographic and clinical characteristics of cases

**Table 1:** Demographic characteristics of cases

Demographics and past medical history	Total number − 36 (%)
Male gender	33 (91.6%)
Age (years)	52 (range 44–66)
Comorbidity	28 (77.7%)
1) Diabetes mellitus	16 (44.5%)
2) Chronic kidney disease	6 (16.6%)
3) Chronic lung disease	2 (5.5%)
4) Chronic liver disease	0
5) Malignancy	2 (5.5%)
6) Solid organ transplantation	2 (5.5%)
COVID-19	32 (88.8%)
Corticosteroid	34 (94.4%)
Anakinra or tocilizumab	20 (55.5%)
Cancer chemotherapy	2 (5.5%)
Surgery within 30 days prior to hospitalization	0
In ICU (Intensive Care Unit) at the time of candidemia	35 (97%)
Mechanical ventilation	32 (88.9%)
Central line	35 (97%)
Hemodialysis	9 (25%)
Extracorporeal membrane oxygenation (ECMO)	5 (14%)
Isolation of *Candida* species from ≥ 2 non-sterile sites before development of candidemia	23 (64%)
Isolation of *Candida auris* before development of candidemia	19 (52.7%)

The first case of *Candida auris* blood stream infection in Qatar was detected on March 2020. The patient was a 55 years old previously healthy Indian male admitted to Intensive Care Unit due to type 1 respiratory failure caused by H1N1 influenza pneumonia requiring ECMO support. He had *C. auris* colonization of the respiratory tract and urinary catheter from 2 weeks prior to the diagnosis of candidemia. The source of candidemia was suspected clinically from arterial line, which was removed, and he was treated with anidulafungin to which the organism was sensitive. He cleared *C. auris* from blood in 4 days. Anidulafungin was continued for 2 weeks, and he was successfully shifted to a medical ward within 1 month. There was a total of 36 patients with *C. auris* blood stream infection in the study period, 33 of them were males (Table 1). The median age was 52 years (range 44–66). All but one patient was in the intensive care unit. 32 patients (88.8%) were admitted to ICU for severe COVID-19 infection. Cases were distributed among six different facilities with half of the cases from Hazm Mebaireek General Hospital which was the main hospital assigned for patients with COVID-19 requiring intensive care (Hazm Mebaireek General Hospital − 19, Hamad General Hospital- 9, Dukhan hospital − 3, AlWakra hospital – 3, Qatar Rehabilitation Institute − 1, and Rumaila hospital − 1). The patient who was not in ICU at the time of candidemia was a case of end-stage renal disease undergoing regular hemodialysis through a permanent catheter. This patient had also undergone a craniectomy for an intracranial bleed and had a complicated post-operative course, requiring an extended stay in the ICU. The candidemia occurred just four weeks after the patient was shifted from the ICU. Only 4 patients had significant immunosuppression before development of COVID-19 pneumonia (two cases each of solid organ transplantation and malignancy). Other demographic characteristics are mentioned in Table 1. 16 patients were diabetic with ten of them having HbA1c above 8% at admission to ICU. The median length of stay in ICU before development of candidemia was 37 days (range 14–102). 89% of the patients were undergoing mechanical ventilation. The median Sequential Organ Failure Assessment (SOFA) Score on day 1 of candidemia was 6 (Range 2–16). 30 out of the 36 patients had other hospital acquired resistant organisms isolated from various clinical samples, including Methicillin resistant *Staphylococcus aureus (*MRSA*)* in 13.8% of cases, Multidrug resistant (MDR) *Enterobacterales* in 25%, MDR *Pseudomonas aeruginosa* in 25%, *Stenotrophomonas maltophilia* in 41.6%, *Acinetobacter* in 8.3% and *Elizabethkingia* in 13.8% of cases. *Stenotrophomonas maltophilia* was the multidrug resistant bacteria most often isolated (41.6%); Multidrug resistant *P. aeruginosa* was the commonest organism with acquired resistance. 52% of the patients had *C. auris* isolated before candidemia (routine screening culture positive in 13, urine culture in 4, and respiratory sample in 2 cases). Central line was a possible source of candidemia in 30 patients, which was removed in 24 (median of 2 days before removal, range 1–7). Other identified sources of candidemia were urinary catheter [1], pelvic collection [1], and secondary peritonitis [1]. Source was unidentified in 3 patients. Three patients had recurrence of *C. auris* candidemia in the 30 day follow up period: one case each secondary to fungal peritonitis, undrained pelvic collection, and recurrence of Central line associated blood stream infection (CLABSI).

### Anti-fungal susceptibilities

**Table 2:** Antifungal susceptibilities of 33 *Candida auris* isolates from bloodstream infection

Antifungal drug	ECV* (µg/mL)	Tentative MIC breakpoints** (µg/mL)	No. of susceptible/ wild-type isolates/total tested (% susceptibility)	Median MIC (µg/mL)	MIC range (µg/mL)
Amphotericin B	N/A***	≥ 2	5/33 (15.2%)	2	0.5-4
Fluconazole	N/A	≥ 32	3/33 (9%)	128	16–128
Itraconazole	N/A	N/A		0.25	0.06-16
Posaconazole	N/A	N/A		0.06	0.015-8
Voriconazole	N/A	N/A		0.5	0.12-8
Anidulafungin*	1	≥ 4	28/28 (100%) by both ECV and tentative breakpoints	0.12	0.06-1
Caspofungin*	0.5	≥ 2	30/33 (91%) by both ECV and tentative breakpoints	0.25	0.12-8
Micafungin*	0.5	≥ 4	33/33 (100%) by both ECV and tentative breakpoints	0.12	0.06–0.5
Flucytosine	N/A	N/A		0.12	0.06-64

*Epidemiological Cutoff Value-CLSI [13]

**Centers for Disease Control and Prevention [14]

***Not available

Antifungal susceptibilities are shown in Table 2. Information on antifungal susceptibilities was unavailable for three isolates. Twenty- eight isolates were tested using the Sensititre™ YeastOne™ YO9 AST Plate (Thermo Fisher Scientific, Carlsbad, U.S.A) and five isolates using the VITEK® AST- YS09 Test Card on the VITEK® 2 (BioMérieux, Marcy-l’Étoile, France). Anidulafungin, posaconazole and itraconazole were not available on the VITEK® AST- YS09 Test Card so the susceptibility could not be determined for the five isolates tested by this method.

A wide variation in MIC ranges was noted (see Table 2) for the second generation triazoles such as voriconazole and posaconazole, itraconazole and flucytosine for which there are currently no CLSI ECVs or CDC tentative breakpoints; some isolates showed very low MICs against these agents. For example, MICs were at least 1-2-fold dilutions lower than the median for 35.7% (ten) of isolates for itraconazole, 57.1% (sixteen) of isolates for posaconazole, 15% (five) of isolates for voriconazole and 25.8% (eight) of isolates for flucytosine.

### Risk factors

**Table 3:** Factors related to 30-day mortality rate in patients with *Candida auris* candidemia

Risk factors	Total number	Number of death (percentage)	Odds ratio (95% confidence interval)	P value
Age > 65	10	5 (50%)	1.36 (0.31–5.89)	0.67
Hba1c > 8	10	5 (50%)	1.36 (0.31–5.89)	0.67
Hemodialysis	9	6 (66.6%)	3.4 (0.69–16.6)	0.12
Extracorporeal membrane oxygenation (ECMO)	4	5 (80%)	6.3 (0.63–63.6)	0.08
Source control not attained	10	7 (70%)	4 (0.80-19.81)	0.078
Persistent candidemia > 5 days	8	6 (75%)	5.4 (0.91–31.93)	0.04
Sequential Organ Failure Assessment (SOFA) score ≥ 9	12	11 (92%)	41 (4.3–405)	0.00005

### Treatment and outcome

Echinocandins were the antifungal given in all patients (caspofungin in 4 cases and anidulafungin in the rest). Voriconazole was added to two patients who required prolonged antifungal treatment. Flucytosine and voriconazole were added in another case. Out of all the patients who died within 30 days, only two did not receive antifungal treatment as their culture results arrived post death. The median antifungal duration was 14 days (range 1-100). 15 patients (41.6%) died within 30 days of isolation of *C. auris* from blood, and 8 of them died within 7 days. Factors associated with mortality are mentioned in Table 3. Among the 30 cases of central line-associated candidemia, 24 had their central line promptly removed (median time from detection to removal- 2 days). Among these patients, 16 survived on day 30. Conversely, among the patients whose central line was not removed, only one patient survived on day 30. Three patients had recurrence of candidemia in 30 days, one of them expired. One patient with fungal peritonitis and another with pelvic collection had sources that were inadequately controlled. The source in the third patient was central line.

## Discussion

The most clinically important *Candida auris* invasive infection is blood stream infection, with a mortality rate ranging from 30 to 60% [15–18]. In a hospital outbreak of *C. auris* candidemia, it was identified as the sixth most common cause of blood stream infection, reaching up to 7.38 infections per 1000 admissions [18]. According to a study conducted between August 2020 and January 2021 in the intensive care units of two hospitals in India, there was a twofold increase in candidemia cases among individuals with COVID-19 compared to those without COVID-19, with *Candida auris* as the predominant species, accounting for 42% of these cases [19]. Intriguingly, countries that had not previously recorded this yeast have now observed COVID-19-associated *C. auris* candidemia [20].

The mortality of 41.6% in our study cohort is on par with the other published literature [2, 16, 21, 22]. Two studies conducted in the neighboring country Oman reported similar fatality rates of 52.5% and 53.1% among patients admitted to the ICU with invasive *C. auris* [23, 24]. Another study by Mohsin et al. from Oman found a mortality rate of 39.1% [21]. Additionally, a study by Khan et al. from Kuwait reported a mortality rate of 60% for invasive *Candida auris* infection [25].

Recurrence was noted only in 3 patients, two of them had probable deep focus of infection inadequately controlled and in the third patient it was a second episode of CLABSI. Recurrence of *C. auris* infection is well known. In a tertiary care hospital outbreak in Spain during 2016–2017, it was observed that 14.6% of patients experienced a recurrence of candidemia, while 29.2% of patients had persistent candidemia [26]. It has also been observed that patients with *C. auris* candidemia have an increased risk of microbiologic recurrence within 60 days after completing antifungal therapy compared to other *Candida* spp [27]. It has been noted in previous case reports that recurrent infection can contribute to development of resistance [28, 29].

89% of our cases had severe COVID-19 infection as the reason for ICU admission. The occurrence of invasive *Candida* infections in COVID-19 patients in ICUs was noted early during the pandemic [30]. The high number of COVID-19 patients requiring ICU care, prolonged hospital stays, the use of central venous catheters, systemic corticosteroid therapy, and broad-spectrum antibiotics increase the risk of invasive candidiasis [30–32]. Moreover, the strain on healthcare workers and overcrowding during the pandemic may impede infection control measures [33]. *Candida auris* infections linked to COVID-19 patients have been reported globally, including the USA, India, Pakistan, and Oman [34, 35]. Out of the 4 patients of *C. auris* candidemia who didn’t have COVID-19 infection, the 30-day mortality was 50%.

The propensity of co-infection with multidrug-resistant pathogens in COVID-19 cases explains why 83% of our patients were affected by hospital-acquired resistant pathogens. According to a comprehensive systematic review and meta-analysis, the pooled prevalence of co-infection with multidrug resistant bacteria and fungal organisms in COVID-19 patients was estimated to be 24% (95% CI 8–40%; n = 25 studies: I2 = 99%) and 0.3% (95% CI 0.1–0.6%; n = 8 studies: I2 = 78%), respectively [36]. In a study conducted by Omrani et al., it was found that in COVID-19-associated candidemia cases in the ICU, the co-infection rate with *Enterobacterales* was 26.3%, *Stenotrophomonas maltophilia* was 18.8%, and multidrug-resistant *Pseudomonas species* was 10% [37].

SOFA score above 9 had statistically significant association with mortality, as depicted in many other studies of candidemia [37–40]. Among patients with SOFA score below 9, only one patient died within 7 days. Other significant associations with mortality are persistent candidemia of more than 5 days and lack of source control (Table 3).

*C. auris* is a human threat due to its intrinsic resistance to one or more classes of antifungal agents [3, 6, 41]. Resistance to fluconazole, amphotericin B, caspofungin, and anidulafungin has been reported in the metanalysis by Chen et al., as 91%, 12%, 12.1% and 1.1% respectively [2].

Echinocandins: All our isolates were broadly sensitive to the echinocandins, with 100% susceptibility to anidulafungin and micafungin and a slightly reduced but still acceptable susceptibility of 91% to caspofungin. There is known to be significant interlaboratory variability in the determination of in vitro caspofungin MICs using the CLSI recommended reference methods that has contributed to reports of false resistance [12]. As the commercial assays we used are similar to the CLSI broth microdilution methods and as all three caspofungin-resistant isolates were susceptible to micafungin and anidulafungin, this was highly suggestive of false positive resistance.

Laboratories that only test for caspofungin MICs should therefore undertake confirmatory testing using either micafungin or anidulafungin [13] for intermediate or resistance isolates; both are more reliable predictors of actual echinocandin resistance. Though the tentative breakpoints for echinocandins proposed by the CDC are at least two-fold dilutions higher than the more recently published CLSI ECVs, we had no isolates whose MICs fell between the CDC breakpoints and CLSI ECVs- our isolates either had MICs that were within the CLSI ECV or were higher than the CDC breakpoints. However, isolates with these ‘in-between’ MICs would pose a challenge in terms of laboratory interpretation of susceptibility and its application to patient management. Echinocandins are probably best avoided for these isolates if other suitable anti-fungal drugs are available. However, it may be prudent to combine echinocandins with another anti-fungal drug from a different class such as a second-generation azole where they are being considered for treatment.

Amphotericin B: Unlike other published literature our rate of amphotericin B resistance is high, almost 85%. In the meta-analysis published by Chen et al., the amphotericin B resistance rate was 12% [2] and in the systematic review by Osei et al., it was 15.4% [22]. In the United States, about 90% of *C. auris* isolates are resistant to fluconazole which is similar to our study findings, about 30% are resistant to amphotericin B, and less than 5% have been resistant to echinocandins [12]. The reported rates of resistance to fluconazole and amphotericin B respectively in neighboring Gulf Cooperation Council (GCC) countries are as follows: Saudi Arabia [42]- 87.5% and 62.5% resistance, Kuwait [43]- 100% and 23.2% resistance, and Oman [23]- 58.3% and 33.3% resistance. The reasons for the somewhat higher resistance rates to amphotericin B within Qatar are currently unclear. The *C. auris* isolates found in Qatar, as well as in Kuwait, Oman, UAE, and Saudi Arabia, all belong to the South Asian Clade I [10], though the Iranian Clade 5 has been isolated from the Islamic Republic of Iran which is a close geographically [18, 19].In the study conducted by Fatma et al. on the molecular characterization of outbreak isolates in patients with COVID-19 within Qatar, it was discovered that over 70% (22 out of 28) of the *C. auris* isolates exhibited resistance to both fluconazole and amphotericin B. Their molecular data provided evidence of the emergence of multidrug-resistant (MDR) strains within the country, containing novel mutations associated with increased resistance to azoles, echinocandins and amphotericin B [10]. However, the molecular mechanism of resistance to amphotericin B was not well understood; they found one isolate resistant to three classes of anti-fungal drugs, which had a unique premature stop codon in ERG3 and novel mutations in CDR2, which they suggest may be associated with elevated amphotericin B and azole resistance. Future research focusing specifically on the molecular characterization of amphotericin B resistant *C. auris* isolates from the State of Qatar would be very beneficial.

Azoles: Despite the lack of data on breakpoints and ECVs for the second generation triazoles, itraconazole (first generation azole) and flucytosine, some isolates had MICs that were very low and comparable to breakpoint values for other *Candida* species [12]. Though extrapolation of these values to *C. auris* is difficult in the absence of any supporting evidence, it does suggest that these agents could be considered for patients where echinocandins cannot be used and /or an additional agent is being considered e.g., persistent candidemia. We would agree with the CDC that the decision to treat with voriconazole or other second generation triazoles should be made on a case-by-case basis as isolates resistant to fluconazole may occasionally respond to these agents [14]. We successfully treated 2 patients with persistent candidemia by combination therapy with anidulafungin and voriconazole and one case with addition of both flucytosine and voriconazole.

## Conclusion

Mortality is high in our cohort of *C. auris* blood stream infections, with a recurrence rate close to 10%. Most of our cases were COVID-19 associated. Management of infection should focus on reducing the impact of statistically significant factors that increase mortality; better source control and early institution of additional anti-fungal drugs for persistent candidemia should improve patient outcomes. Local resistance to amphotericin B is high- more research is needed to understand the reasons for this. Echinocandins are the treatment of choice for empiric therapy; laboratories that test only caspofungin should carry out confirmatory testing using either micafungin or anidulafungin for intermediate or resistant isolates. The loss of amphotericin B as a viable treatment option in many cases has a negative impact on patient outcomes for infections at sites where echinocandin penetration is poor e.g., central nervous system, ophthalmic, and urinary infections [44].

## Limitations

Being a medical record based retrospective review, there are several limitations for the study including incomplete or missing data, confounding factors, lack of a control group, and the potential for recall bias. Data quality and completeness may vary, leading to missing information. Absence of a control group makes it challenging to establish causality or determine the true effects of the infection. Additionally, the study’s findings may not be generalizable to broader populations or healthcare settings, and temporal ambiguity may exist in the collected data. Molecular characterization of isolates was not undertaken as it was outside the remit of the study so any genetic basis for resistance could not be elucidated.

## Data Availability

The datasets used and/or analysed during the current study are available from the corresponding author on reasonable request.
